# Identifying Barriers to Enrollment in Patient Pregnancy Registries: Building Evidence Through Crowdsourcing

**DOI:** 10.2196/30573

**Published:** 2022-05-25

**Authors:** Jeanne M Pimenta, Jeffery L Painter, Kim Gemzoe, Roger Abramino Levy, Marcy Powell, Paige Meizlik, Gregory Powell

**Affiliations:** 1 GlaxoSmithKline Brentford, Middlesex United Kingdom; 2 Safety Innovation and Analytics GlaxoSmithKline Durham, NC United States; 3 GlaxoSmithKline Stevenage United Kingdom; 4 GlaxoSmithKline Collegeville, PA United States

**Keywords:** belimumab, crowdsourcing, systemic lupus erythematosus, pregnancy, registry

## Abstract

**Background:**

Enrollment in pregnancy registries is challenging despite substantial awareness-raising activities, generally resulting in low recruitment owing to limited safety data. Understanding patient and physician awareness of and attitudes toward pregnancy registries is needed to facilitate enrollment. Crowdsourcing, in which services, ideas, or content are obtained by soliciting contributions from a large group of people using web-based platforms, has shown promise for improving patient engagement and obtaining patient insights.

**Objective:**

This study aimed to use web-based crowdsourcing platforms to evaluate Belimumab Pregnancy Registry (BPR) awareness among patients and physicians and to identify potential barriers to pregnancy registry enrollment with the BPR as a case study.

**Methods:**

We conducted 2 surveys using separate web-based crowdsourcing platforms: Amazon Mechanical Turk (a 14-question patient survey) and Sermo RealTime (a 11-question rheumatologist survey). Eligible patients were women, aged 18-55 years; diagnosed with systemic lupus erythematosus (SLE); and pregnant, recently pregnant (within 2 years), or planning pregnancy. Eligible rheumatologists had prescribed belimumab and treated pregnant women. Responses were descriptively analyzed.

**Results:**

Of 151 patient respondents over a 3-month period (n=88, 58.3% aged 26-35 years; n=149, 98.7% with mild or moderate SLE; and n=148, 98% from the United States), 51% (77/151) were currently or recently pregnant. Overall, 169 rheumatologists completed the survey within 48 hours, and 59.2% (100/169) were based in the United States. Belimumab exposure was reported by 41.7% (63/151) patients, whereas 51.7% (75/145) rheumatologists had prescribed belimumab to <5 patients, 25.5% (37/145) had prescribed to 5-10 patients, and 22.8% (33/145) had prescribed to >10 patients who were pregnant or trying to conceive. Of the patients exposed to belimumab, 51% (32/63) were BPR-aware, and 45.5% (77/169) of the rheumatologists were BPR-aware. Overall, 60% (38/63) of patients reported belimumab discontinuation because of pregnancy or planned pregnancy. Among the 77 BPR-aware rheumatologists, 70 (91%) referred patients to the registry. Concerns among rheumatologists who did not prescribe belimumab during pregnancy included unknown pregnancy safety profile (119/169, 70.4%), and 61.5% (104/169) reported their patients’ concerns about the unknown pregnancy safety profile. Belimumab exposure during or recently after pregnancy or while trying to conceive was reported in patients with mild (6/64, 9%), moderate (22/85, 26%), or severe (1/2, 50%) SLE. Rheumatologists more commonly recommended belimumab for moderate (84/169, 49.7%) and severe (123/169, 72.8%) SLE than for mild SLE (36/169, 21.3%) for patients trying to conceive recently or currently pregnant. Overall, 81.6% (138/169) of the rheumatologists suggested a belimumab washout period before pregnancy of 0-30 days (44/138, 31.9%), 30-60 days (64/138, 46.4%), or >60 days (30/138, 21.7%).

**Conclusions:**

In this case, crowdsourcing efficiently obtained patient and rheumatologist input, with some patients with SLE continuing to use belimumab during or while planning a pregnancy. There was moderate awareness of the BPR among patients and physicians.

## Introduction

### Background

Data on the safety profile of novel therapies before, during, and soon after pregnancy are of paramount importance to patients and their treating physicians. However, clinical trials before regulatory approval generally exclude pregnant women, require the use of a highly effective contraception method, and require withdrawal of treatment if a pregnancy is identified during the relevant period of exposure. Basic pregnancy outcome information is actively sought when pregnancy occurs while in a clinical trial [1]. As a result, physicians may be reluctant to prescribe novel treatments to pregnant women owing to a lack of human pregnancy data to allow an informed benefit-to-risk balance decision. To generate additional safety data in pregnant women, postapproval studies using data collected from pregnancy registries can be undertaken [1]. Voluntary pregnancy registries enroll pregnant patients who have received a therapy of interest into a cohort, with nonexposed pregnant women being an ideal comparator group, although this is not always feasible [1]. Registries can generate timely and comprehensive data on the maternal and fetal safety profile of a specific drug, including postnatal outcomes [1].

Recruitment of patients to pregnancy registries is often challenging [1-5]. Enrollment during the early years of a pregnancy registry can be very low, with reasons including the drug of interest being new to the market, the voluntary enrollment process often used, and the therapy of interest being rarely prescribed to pregnant women [1-5]. Registries may use a variety of approaches to maximize enrollment, such as designating a single coordinating center to handle recruitment, or using automated alerts of pregnancy registrations [1,3]; however, recruitment challenges remain.

Systemic lupus erythematosus (SLE) is a chronic multisystem autoimmune disease that predominantly affects women, many of whom are of childbearing age [6-8]. Patients with SLE with a history of active lupus nephritis or antiphospholipid antibodies during pregnancy are susceptible to premature birth and/or hypertension, highlighting the need for prepregnancy counseling [9]. Patients with autoantibody-positive active SLE may be prescribed belimumab, a B-lymphocyte stimulator inhibitor [10,11]. The Belimumab Pregnancy Registry (BPR; GlaxoSmithKline study 114256; NCT01532310) is a multinational, prospective, voluntary registry [12] established to document belimumab exposure in pregnant women. The BPR was initiated in 2012 with the aim of recruiting 500 pregnant women treated with belimumab; however, the number of patients enrolled has been low (69 evaluable patients as of July 10, 2020) despite considerable awareness-raising activities.

### Objectives

Crowdsourcing, in which a large group of people is tasked either competitively or noncompetitively to solve a problem or complete a task on web, has shown promise across several areas of health [13,14]. Several studies have used crowdsourcing to improve patient engagement and obtain patient insights to help inform future research directions [14,15]. Crowdsourcing platforms such as Amazon Mechanical Turk (MTurk) [16] allow researchers to easily identify platform members who meet specific requirements, such as those with a specific disease or receiving particular treatments [17]. This study used 2 web-based crowdsourcing platforms to evaluate awareness of the BPR among patients and physicians and identify potential barriers to enrollment in pregnancy registries by using the BPR as a case study example.

## Methods

### Project Design

Two surveys were designed and conducted using 2 separate web-based crowdsourcing platforms: MTurk [16] and Sermo RealTime [18].

Patient surveys were conducted via MTurk [16], which enables the delivery of tasks (human information tasks) to a globally distributed, quality-managed workforce (Turkers) who self-select their participation in the survey. Turkers were remunerated as per Amazon guidelines [19] for completion of the questionnaires, with remuneration amounts set in line with the GlaxoSmithKline policy to ensure that payment rates are similar to national minimum wage standards. Surveys were anonymous and quick, but it was not possible to verify the self-reported disease status and demographics of Turkers, and follow-up questions after the initial response were not possible. Self-imposed best practices were used to maximize the quality of results. This included the rejection of incomplete surveys and surveys from respondents who had previously reported inconsistent demographics, such as gender. Survey submission time was also inspected and surveys that took <30 seconds to complete were rejected. Respondents were not financially compensated if their survey was rejected, and these responses were removed from the data set. Rheumatologist surveys were conducted via Sermo RealTime [18], a medical crowdsourcing site that has >800,000 verified, licensed physician members worldwide. Sermo is a commercially available fee-for-service platform on which surveys are anonymous and quick. Follow-up questions were made possible after the initial response.

Patient and rheumatologist survey questions were in the English language only and were designed to assess belimumab exposure during pregnancy, patient and rheumatologist awareness of the BPR, rheumatologist willingness to refer patients to the BPR, perceived BPR enrollment barriers for physicians and patients, and SLE treatment during pregnancy. The patient survey consisted of 14 questions and the rheumatologist survey consisted of 11 questions (2 screening questions, followed by 9 questions). Responses were collected from patients and rheumatologists in eight (Austria, Belgium, Canada, France, Germany, Spain, Sweden, and United States) of the countries the BPR was active in, which were deemed to have sufficient MTurk and Sermo populations to provide a useful number of responses. The patient and rheumatologist survey questions are available in Multimedia Appendix 1.

### Patient Population

Eligibility was assessed using a short set of screening questions in which patients could self-report their demographic and clinical characteristics. In addition, the Amazon Turk selection criteria were used to request access to the survey only for women from the 8 countries where the BPR was active. Patients who reported that they were women; aged 18-55 years; had a diagnosis of SLE; and were pregnant, had recently been pregnant (within 2 years), or were planning a pregnancy were eligible for inclusion. Patients completed their web-based MTurk questionnaire regarding SLE medication use and BPR awareness. The survey was conducted for 3 months, from June to September 2018.

### Rheumatologist Population

The rheumatologist survey targeted rheumatologists (in the 8 countries where the survey was active) based on the Sermo specialty classifications, who had a history of prescribing belimumab in pregnancy. A target of 200 rheumatologist respondents was set, with the availability of the questionnaire determined by the number of responses received. Rheumatologists completed a web-based questionnaire on prescription patterns and factors related to BPR awareness and enrollment. Responses were obtained within a 48-hour period in September 2018.

### Analysis

As there was no direct way to validate whether Turkers had SLE, the similarity between rheumatologist-recommended and patient self-reported treatment options according to SLE severity were compared in patients who were pregnant or trying to conceive using a set of questions regarding medication use. Chi-square statistics were computed for each individual drug per SLE severity level, as well as an overall chi-square statistic to determine how well the 2 groups aligned and to help determine the reliability of the patient responses received on MTurk. The null hypothesis was that the proportion of medication use across the 6 treatment options reported by patients was at the same rate as that recommended by rheumatologists. All other responses were descriptively analyzed.

### Ethics Approval

According to UK Health Research Authority criteria [20], this project did not require ethical approval. The project was conducted under GlaxoSmithKline plc.’s Scientific Engagement Policy [21] which enables the non-promotional interaction and exchange of scientific information between GlaxoSmithKline plc. and external communities to advance scientific and medical understanding and improve patient care. Patients, general practitioners and practice managers all provided consent to take part in the two crowdsourcing platforms via signed consultancy contracts and were reimbursed for their time at Fair Market Value.

## Results

### Participant Demographics and Clinical Characteristics

#### Patients

A total of 151 patients, primarily from the United States (n=148, 98%), responded with a steady submission response rate over a 3-month period (Figure S1 in Multimedia Appendix 2). The average time to complete the survey was 9 minutes. Most patients were aged between 26 and 35 years and reported mild or moderate SLE. Half of the patients (77/151, 51%) were either currently or recently pregnant (Table 1).

**Table 1:** Patient and rheumatologist demographics and characteristics.

Demographics				Respondents
**Patients (N=151)**				
	**Age (years), n (%)**			
		18-25	26 (17.2)	
		26-35	88 (58.3)	
		36-45	32 (21.2)	
		46-55	5 (3.3)	
	**SLE^a^** **disease severity, n (%)**			
		Mild	64 (42.4)	
		Moderate	85 (56.3)	
		Severe	2 (1.3)	
	**Pregnancy status, n (%)**			
		Currently pregnant	23 (15.2)	
		Recently pregnant	54 (35.8)	
		Planning pregnancy	74 (49)	
	**Country, n (%)**			
		United States	148 (98)	
		Canada	1 (0.7)	
		Estonia	1 (0.7)	
		Venezuela	1 (0.7)	
**Rheumatologists (N=169)**				
	**Country, n (%)**			
		United States	100 (59.2)	
		Germany	33 (19.5)	
		Canada	10 (5.9)	
		France	10 (5.9)	
		Spain	10 (5.9)	
		Austria	2 (1.2)	
		Belgium	2 (1.2)	
		Sweden	2 (1.2)	
	Length of time treating patients with SLE (years), mean (range)			12.2 (1-33)
	**Practice setting, n (%)**			
		Hospital	34 (20.1)	
		Academic medical center	43 (25.4)	
		General primary care	2 (1.2)	
		Private practice	87 (51.5)	
		Other	3 (1.8)	

^a^SLE: systemic lupus erythematosus.

#### Rheumatologists

A total of 169 rheumatologists completed the survey. Responses were obtained within a 48-hour period from rheumatologists, with most being US-based (100/169, 59.2%; Table 1). Respondents from the United States were geographically well distributed.

### Barriers to Study Enrollment

Overall, 85.2% (144/169) rheumatologists (84/100, 84% in the United States) explained why they would not prescribe belimumab during pregnancy. Concerns among rheumatologists included an unknown pregnancy safety profile (119/169, 70.4% overall; 75/100, 75% in the United States), preference for other treatment options (63/169, 37.2% overall; 39/100, 39% in the United States), the disease being mild or symptoms being tolerable (50/169, 29.6% overall; 27/100, 27% in the United States), and *other* (2/169, 1.2% overall; 0/100, 0% in the United States). In addition, 23.7% (40/169) rheumatologists (28/100, 28% in the United States) reported that their patients had no concerns about belimumab exposure during pregnancy, whereas 16% (27/169; 12/100, 12% in the United States) reported that their patients preferred other treatments, 23.1% (39/169; 21/100, 21% in the United States) reported that their patients had a desire to reduce medication, and 61.5% (104/169; 66/100, 66% in the United States) reported that their patients were concerned about the unknown safety profile of belimumab during pregnancy.

### BPR Awareness

#### Patients

**Figure 1:**
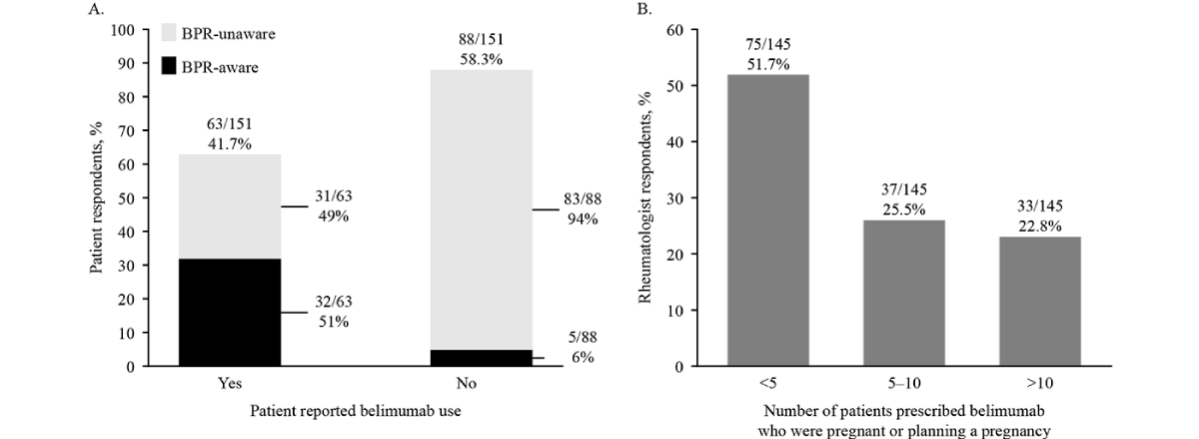
(A) Patient-reported previous belimumab exposure and Belimumab Pregnancy Registry (BPR) awareness and (B) rheumatologist-reported belimumab prescriptions for patients who were pregnant or planning a pregnancy.

**Figure 2:**
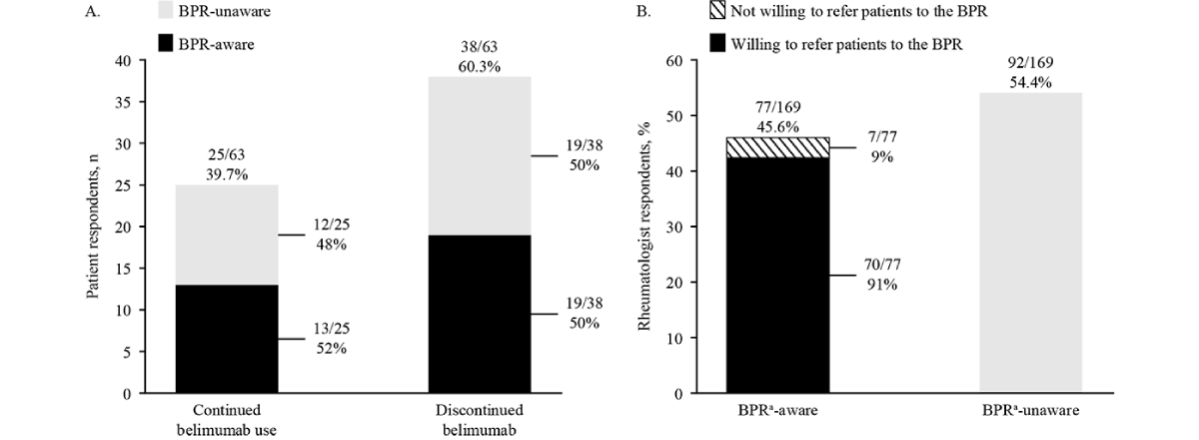
(A) Patient-reported belimumab discontinuation because of pregnancy or planned pregnancy and Belimumab Pregnancy Registry (BPR) awareness and (B) rheumatologist-reported BPR awareness and willingness to refer patients to the BPR among those who were BPR-aware.

Awareness of the BPR was greater in patients who had been previously exposed to belimumab (32/63, 51%) than in those who had never been exposed to belimumab (5/88, 6%; Figure 1A). Among 37 women who reported the source of their awareness of the BPR, the most commonly reported sources of knowledge about the study were rheumatologists (21/37, 57%), followed by a friend or family member (8/37, 22%), internet (4/37, 11%), or brochure (4/37, 11%). Overall, 60% (38/63) patients reported that they discontinued belimumab because of pregnancy or planned pregnancy, whereas 40% (25/63) patients continued treatment during pregnancy or while trying to become pregnant (Figure 2A). For patients who discontinued belimumab, BPR awareness did not appear to affect the decision to continue or discontinue, with approximately equal numbers in the continued and discontinued groups aware or not aware of the BPR; 52% (13/25) patients who continued treatment were aware of the BPR, whereas 48% (12/25) were unaware of the BPR; 50% (19/38) patients who discontinued treatment were aware of the BPR, whereas the other 50% (19/38) patients were unaware of the BPR (Figure 2A).

#### Rheumatologists

Overall, 45.6% (77/169) rheumatologists were aware of the BPR, with most BPR-aware rheumatologists (70/77, 91%) referring patients to the registry (Figure 2B, Multimedia Appendix 3). Among US rheumatologists, 43% (43/100) were aware of the BPR, and most referred patients for BPR enrollment (40/43, 93%).

### Belimumab Exposure

#### Patients

Previous belimumab exposure was reported by 41.7% (63/151) patients (Figure 1A). Belimumab exposure differed slightly among patients who were pregnant or recently pregnant (39/77, 51%) and those planning a pregnancy (24/74, 32%). Among all patients who had been exposed to belimumab, 54% (34/63) reported <1 year of use, 36% (23/63) reported 1-2 years of use, and 10% (6/63) reported >2 years of use.

#### Rheumatologists

Overall, 145 rheumatologists (86 from the United States) provided the number of patients with SLE (who were pregnant or trying to become pregnant) who they had treated with belimumab over the course of their careers; of these, 75 (51.7%) had prescribed belimumab to <5 patients who were either pregnant or trying to become pregnant, 37 (25.5%) had prescribed belimumab to 5-10 patients in this category, and 33 (22.7%) had prescribed belimumab to >10 patients in this category (Figure 1B). Using the minimum and maximum estimates, this approximately equated to a minimum of 600 patients who had been exposed to belimumab during pregnancy, with the upper estimate being at least 1108. The results were similar in the group of rheumatologists from the United States; 58% (50/86) rheumatologists had prescribed belimumab to <5 patients who were either pregnant or trying to become pregnant, 21% (18/86) had prescribed belimumab to 5-10 patients in this category, and 21% (18/86) had prescribed belimumab to >10 patients in this category.

A total of 8 rheumatologists who completed the survey were screened out; 7 (88%) because they did not prescribe belimumab and 1 (13%) because they did not treat pregnant patients or those trying to become pregnant.

### Treatment Patterns

**Figure 3:**
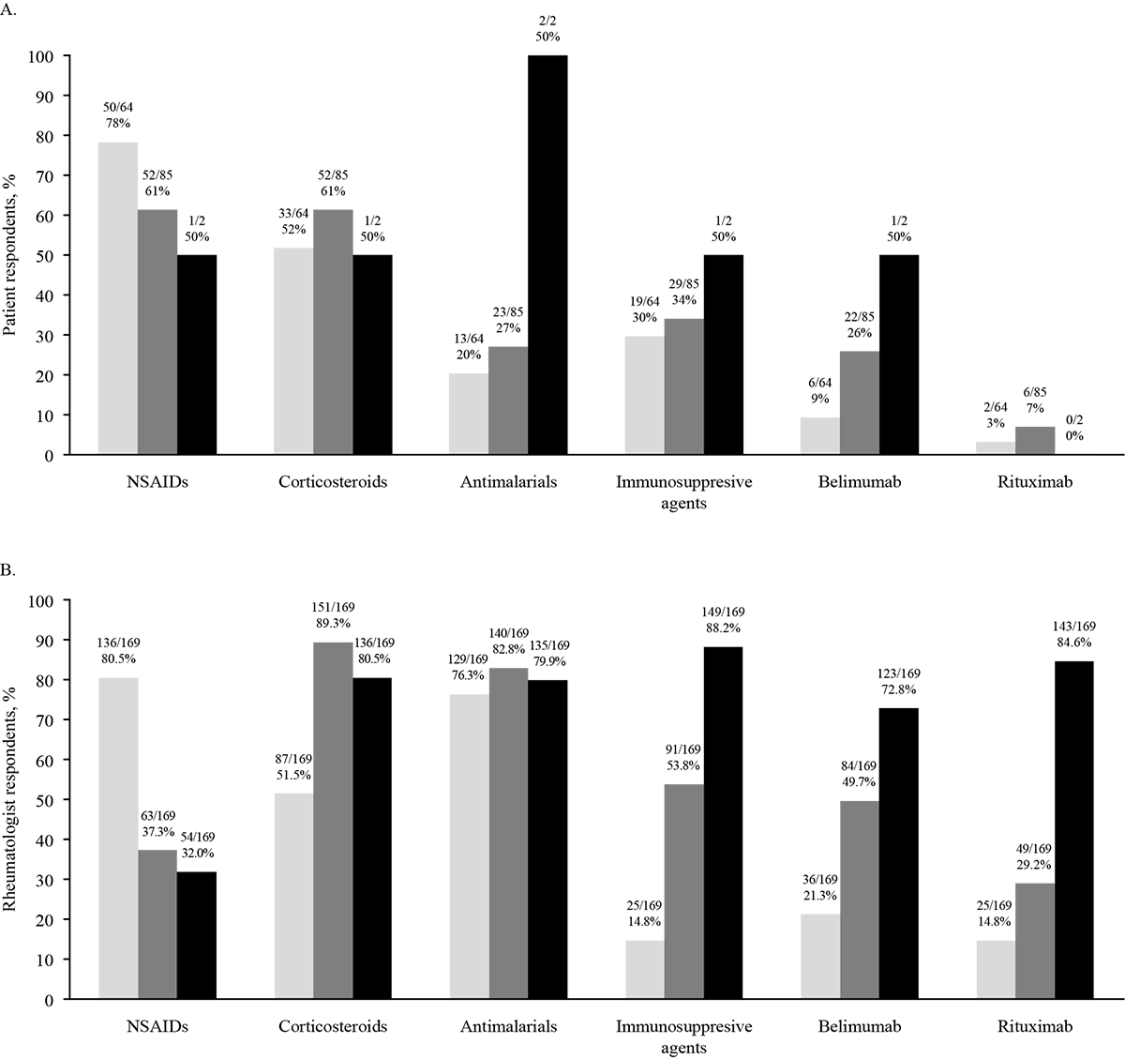
(A) Patient-reported and (B) rheumatologist-recommended treatments during pregnancy or while planning a pregnancy, according to the severity of systemic lupus erythematosus. NSAID: nonsteroidal anti-inflammatory drug.

Belimumab exposure was reported by patients with mild (6/64, 9%), moderate (22/85, 26%), and severe (1/2, 50%) SLE during pregnancy, recently after pregnancy, or while they were trying to conceive (Figure 3A). Rheumatologists more commonly recommended belimumab for patients who were potentially pregnant, recently pregnant, or trying to conceive with moderate (84/169, 49.7% overall; 50/100, 50% in the United States) and severe (123/169, 72.8% overall; 69/100, 69% in the United States) SLE than with mild SLE (36/169, 21.3% overall; 27/100, 27% in the United States; Figure 3B).

Use of immunosuppressive agents during pregnancy, recently after pregnancy, or while trying to conceive was reported by patients with mild (19/64, 30%) or moderate (29/85, 34%), but not severe (1/2, 50%), SLE (Figure 3A). Rheumatologists most commonly recommended immunosuppressive agents for severe disease (149/169, 88.2% overall; 91/100, 91% in the United States), followed by moderate (91/169, 53.8% overall; 56/100, 56% in the United States), and mild (25/169, 14.8% overall; 14/100, 14% in the United States) disease in this group of patients (Figure 3B).

Overall, patient-reported drug exposure by disease severity in patients who were pregnant, recently pregnant, or trying to conceive was statistically different from rheumatologist-recommended treatment options (*P*<.001; Table 2). As only 2 patients self-reported having severe SLE, we further restricted our comparison to only include mild and moderate SLE and found that the null hypothesis must still be rejected (*P*<.001). However, when individual disease severity and treatment options were compared, most of the patient responses (10/18, 56%) agreed with rheumatologists’ recommendations (*P*>.05; Table 2).

**Figure 4:**
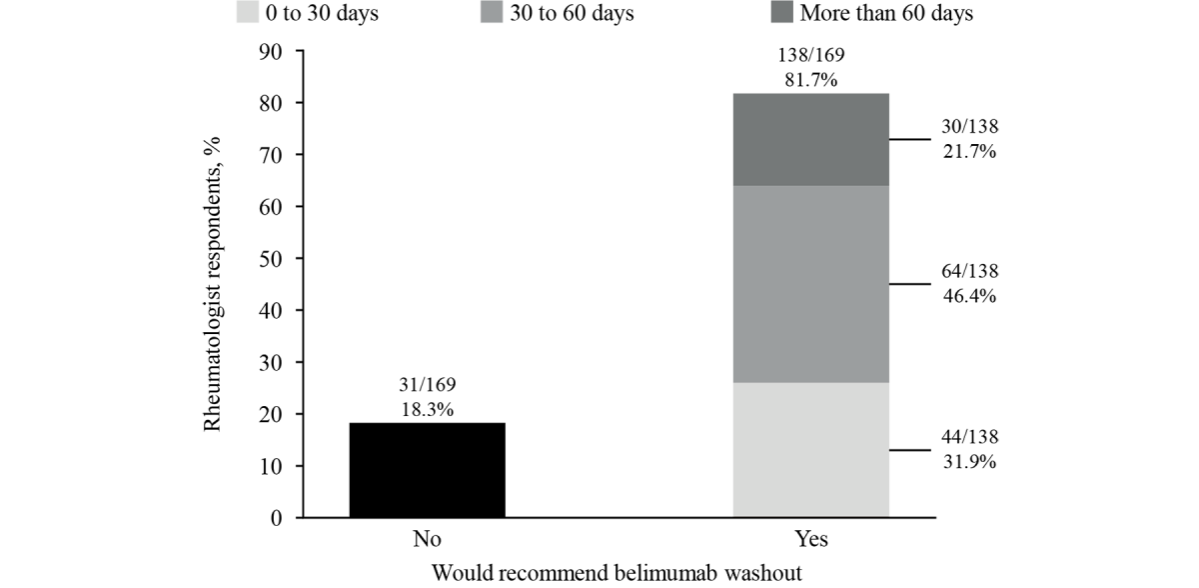
Proportion of rheumatologists recommending belimumab washout before pregnancy and the washout period (days) recommended.

In total, 82% (138/169) of rheumatologists (80/100, 80% in the United States) reported that they would suggest a washout period from belimumab before pregnancy; 31.9% (44/138; 26/100, 26% in the United States) would suggest a period of 0-30 days, 46.4% (64/138; 38/100, 38% in the United States) would suggest a period of 30-60 days, and 21.7% (30/138; 16/100, 16% in the United States) would suggest a period of more than 60 days (Figure 4).

**Table 2:** Chi-square statistics comparing individual drugs per severity level between patient self-reported^a^ treatments and rheumatologist recommendations.

Treatment option	Expected rate of rheumatologist recommendation (%)	Disease severity	Chi-square (*df*)	*P* value	Reject the null hypothesis
NSAID^b^	54	Mild	0.8 (1)	.36	No
Corticosteroids	23	Mild	0.2 (1)	.62	No
Antimalarials	32	Mild	8.8 (1)	.003	Yes
Immunosuppressive agents	9	Mild	0.2 (1)	.67	No
Belimumab	15	Mild	2.1 (1)	.15	No
Rituximab	12	Mild	7.4 (1)	.007	Yes
NSAID	25	Moderate	5.5 (1)	.02	Yes
Corticosteroids	41	Moderate	3.1 (1)	.08	No
Antimalarials	35	Moderate	4.5 (1)	.03	Yes
Immunosuppressive agents	34	Moderate	9.0 (1)	.003	Yes
Belimumab	35	Moderate	9.1 (1)	.003	Yes
Rituximab	23	Moderate	13.7 (1)	.002	Yes
NSAID	21	Severe	5.8 (1)	.02	Yes
Corticosteroids	36	Severe	0.1 (1)	.75	No
Antimalarials	33	Severe	0.7 (1)	.41	No
Immunosuppressive agents	56	Severe	1.1 (1)	.29	No
Belimumab	50	Severe	0.0 (1)	.99	No
Rituximab	65	Severe	1.3 (1)	.25	No
All treatment options by all disease severities	N/A^c^	N/A^c^	73.5 (1)	<.001	Yes

^a^In patients who were pregnant, trying to conceive, or recently pregnant.

^b^NSAID: nonsteroidal anti-inflammatory drug.

^c^N/A: not applicable.

## Discussion

### Principal Findings

Pregnancy registries can provide valuable maternal and fetal safety data for newly available therapies; however, recruitment of patients into these registries can be challenging [1,3,5,22]. In the current project, crowdsourcing was used as a novel method to obtain insights from patients and health care professionals on pregnancy registries, specifically the BPR, a multinational, prospective, voluntary registry, established in 2012 to document belimumab exposure in pregnant women.

Web-based crowdsourcing surveys were completed by a considerable number of patients (N=151) and rheumatologists (N=169), demonstrating that crowdsourcing is an effective data collection method to obtain relevant patient and health care professional inputs into ongoing studies. Crowdsourcing was chosen over traditional surveys as it is cost-effective, rapid, targeted, and provides geographically diverse physician and patient feedback. The crowdsourcing approach allowed for cost-effective specific targeting of women with SLE via the MTurk platform; however, as the Turker population is dynamic, it is important to ensure that an adequate period is provided to reach target response levels. Similarly, Sermo allowed for rapid response rates from physicians, which were typically received within 48 hours in the current project. This analysis adds to the growing body of literature that demonstrates the value of crowdsourcing in health research, with previous studies using crowdsourcing across a range of diagnostic, surveillance, and public health applications, among others [13]. However, drawbacks of a crowdsourcing approach are well known, including potential bias in the sample that reduces the generalizability to a wider population [14,15].

Assessment of the safety of pharmaceutical therapies during pregnancy is necessary to gather data that can be used by health care professionals when treating and counseling patients who are pregnant or who wish to become pregnant [3]. This is of particular importance in patients with diseases such as SLE that predominantly affect women, many of whom are of childbearing age, and can be associated with adverse pregnancy outcomes such as premature birth [23]. The overarching principles of the European League Against Rheumatism to guide the use of SLE medication during pregnancy and lactation include aiming to prevent or suppress maternal disease activity without harm to the fetus or child and balancing the risk of medication for the fetus or child against the risks of untreated maternal disease [24]. The US Food and Drug Administration guidelines on the assessment of the outcomes of pregnancies in women exposed to pharmaceutical products state that pharmacovigilance, pregnancy registries, and complementary data sources can be used to evaluate drug safety during pregnancy [1]. However, pregnant patients are generally a population that is difficult to reach, and the feasibility of there being sufficient treatment exposure and recruitment within the patient population to allow enough reliable pregnancy and infant outcome information to be obtained is a critical consideration in the design of pregnancy registries to ensure a sufficient sample size [1,25]. The European League Against Rheumatism has published recommendations for a core data set that pregnancy registries should aim to collect; by gathering data more uniformly, data from different registry sources could be analyzed together, helping to address the issue of low recruitment [26].

The responses received from patients and rheumatologists in the current project are consistent with previous reports of low recruitment into pregnancy registries despite considerable treatment exposure [2,22]. The results suggest that although some women report belimumab exposure during pregnancy (39/77, 51% of pregnant patient respondents in the current project), very few have entered the BPR to date. This may be because of a lack of awareness, with only approximately 51% (32/63) of patients who had been exposed to belimumab during pregnancy or while planning a pregnancy and 45.6% (77/169) of participating rheumatologists reporting that they were aware of the BPR. These data suggest that pregnancy registries could generally focus on improving engagement levels with both pregnant patients and physicians to help increase awareness and recruitment rates. However, as most patients (21/37, 57%) stated that the source of their BPR awareness was a physician, increasing registry awareness among health care professionals may be a particularly effective method to improve recruitment. This is consistent with previous studies on pregnancy registry recruitment, which also identified physicians as the major target for increasing awareness [2,22].

Interestingly, most rheumatologists (70/77, 91%) who were aware of the BPR reported that they were willing to refer patients to the registry, and almost half of them (70/145, 48.3%) reported that they had prescribed belimumab to >5 pregnant (or soon-to-be pregnant) patients during their career. The relatively high number of rheumatologists who were aware of BPR and willing to prescribe belimumab during pregnancy contrasts with the low recruitment numbers to date. This suggests that there may be additional obstacles that prevent physicians from enrolling patients in pregnancy registries in routine practice. Possibilities include administrative barriers, the voluntary nature of enrollment, the lack of compensation for time and resources, and no immediate benefit to the participating patients. Therefore, recruitment into voluntary patient registries is likely to be reliant on the participation of patient subgroups who may generally be more motivated and health conscious [27].

As expected, low recruitment into pregnancy registries can also be attributed to the current lack of pregnancy safety data for the drug of interest. In this study, only small proportions of rheumatologists and patients reported no concerns about belimumab exposure during pregnancy, and more than half of the rheumatologists (119/169, 70%) and patients (104/169, 62%) reported the unknown pregnancy safety profile of belimumab as a concern. This is consistent with the Food and Drug Administration belimumab prescribing information, which highlights the limited pregnancy data available [11]. Many rheumatologists have also indicated a preference for alternative treatments or a desire to eliminate all treatments during pregnancy. Therefore, further information on the safety profile of belimumab during pregnancy will be of great value to support patient and physician decisions regarding SLE treatment during pregnancy. This can be facilitated by further awareness-raising activities and recruitment of the target population into pregnancy registries or by alternative methods of collecting pregnancy surveillance data.

Survey responses indicated that belimumab treatment is more commonly recommended by rheumatologists as a treatment for patients with SLE who were recently pregnant, potentially pregnant, or trying to conceive with moderate or severe rather than mild disease. In patients who self-reported mild severity, the major drug classes (nonsteroidal anti-inflammatory drugs, corticosteroids, immunosuppressive agents, and belimumab) were all within the expected range of rheumatologist recommendations.

### Strengths and Limitations

The strengths of this project include the size of the sample of patients with SLE and rheumatologists with a history of treating patients with SLE, along with the use of a real-world SLE population with varied self-reported disease severities. Limitations of this analysis include geographic representation being predominantly US-based; thus, country-specific differences in participation rates may influence interpretability or generalizability to other countries outside the United States. Respondents had to have internet access, and there was a bias toward patients with an understanding of the English language given that the survey questions were only available in English. These requirements could have introduced bias regarding the socioeconomic and educational status of the patients, which was not available or requested. Participants were also required to have Amazon MTurk or Sermo accounts and be willing to participate in the survey. Verification that MTurk respondents were female patients with SLE who were pregnant or planning a pregnancy was not possible, and it is possible that some participants completed the survey imposing as patients for financial gain. To mitigate this concern, we implemented screening procedures, such as rejection of incomplete or inconsistent surveys and surveys completed in <30 seconds, to safeguard the validity of the data collected. A review of the strengths and weaknesses of MTurk research found similarities between MTurk participants and traditional samples and concluded that MTurk has many benefits that make it suitable for assessing a variety of behavioral research, with evidence showing that Turkers produce reliable results consistent with standard decision-making biases [28]. In addition, patients had unverified disease severity; reporting mild, moderate, and severe SLE was made at the discretion of MTurk respondents, and although these are likely to be more standardized among rheumatologists, patient perceptions of mild, moderate, and severe disease may differ. We assessed the similarity between rheumatologist-recommended and patient self-reported treatment options according to SLE severity using a set of questions regarding medication use to verify patients’ responses; however, this proxy approach is not without its own limitations given the disproportionate participation between countries. Moreover, rheumatologists’ views on prepregnancy washout were collected for belimumab and not for other treatments. Finally, all data including belimumab exposure were reported by the patients and rheumatologists themselves and could not be verified.

### Conclusions

Web-based crowdsourcing is a viable approach for obtaining patient and physician input and enables insights to be gathered from difficult-to-recruit populations. Using our case example, crowdsourcing responses from patients and rheumatologists suggest that there exists a population of patients with SLE who continue to use belimumab during pregnancy. There was moderate awareness of the BPR among patients and physicians. In contrast, enrollment in the BPR is low despite considerable time and resources being devoted to raising awareness among patients and rheumatologists, as well as a willingness among rheumatologists to refer patients to the registry. Barriers to enrollment in pregnancy registries such as the BPR may include a lack of awareness, preference for alternative or no treatment during pregnancy, lack of data on the benefit/risk profile associated with treatment during pregnancy, and the voluntary nature of the study. Alternative approaches to enrolling patients in pregnancy registries should be explored.

## Appendix

Multimedia Appendix 1 Patient questionnaire.

Multimedia Appendix 2 Recruitment over time via the Amazon Mechanical Turk platform.

Multimedia Appendix 3 Rheumatologists’ reasons for not referring patients to the belimumab registry.

## Abbreviations

BPR: Belimumab Pregnancy Registry
GSK: GlaxoSmithKline
MTurk: Mechanical Turk
SLE: systemic lupus erythematosus

## Data Availability

GlaxoSmithKline (GSK) is committed to publicly disclosing the results of the GSK-sponsored clinical research that evaluates GSK medicines, and as such was involved in the decision to submit. Researchers can inquire about the availability of data from GSK clinical studies that are not listed on the site before they submit a research proposal from ClinicalStudyDataRequest.com.

## References

[1] (2019). Postapproval pregnancy safety studies: guidance for industry: draft guidance. U.S. Food & Drug Administration.

[2] Sinclair S, Cunnington M, Messenheimer J, Weil J, Cragan J, Lowensohn R, Yerby M, Tennis P (2014). Advantages and problems with pregnancy registries: observations and surprises throughout the life of the International Lamotrigine Pregnancy Registry. Pharmacoepidemiol Drug Saf.

[3] Gliklich RE, Dreyer NA, Leavy MB (2014). Registries for Evaluating Patient Outcomes: A User's Guide. 3rd edition.

[4] Krueger WS, Anthony MS, Saltus CW, Margulis AV, Rivero-Ferrer E, Monz B, Hirst C, Wormser D, Andrews E (2017). Evaluating the safety of medication exposures during pregnancy: a case study of study designs and data sources in multiple sclerosis. Drugs Real World Outcomes.

[5] Sarker A, Chandrashekar P, Magge A, Cai H, Klein A, Gonzalez G (2017). Discovering cohorts of pregnant women from social media for safety surveillance and analysis. J Med Internet Res.

[6] D'Cruz DP, Khamashta MA, Hughes GR (2007). Systemic lupus erythematosus. Lancet.

[7] Lau CS, Mak A (2009). The socioeconomic burden of SLE. Nat Rev Rheumatol.

[8] Weckerle CE, Niewold TB (2011). The unexplained female predominance of systemic lupus erythematosus: clues from genetic and cytokine studies. Clin Rev Allergy Immunol.

[9] Smyth A, Oliveira GH, Lahr BD, Bailey KR, Norby SM, Garovic VD (2010). A systematic review and meta-analysis of pregnancy outcomes in patients with systemic lupus erythematosus and lupus nephritis. Clin J Am Soc Nephrol.

[10] Baker KP, Edwards BM, Main SH, Choi GH, Wager RE, Halpern WG, Lappin PB, Riccobene T, Abramian D, Sekut L, Sturm B, Poortman C, Minter RR, Dobson CL, Williams E, Carmen S, Smith R, Roschke V, Hilbert DM, Vaughan TJ, Albert VR (2003). Generation and characterization of LymphoStat-B, a human monoclonal antibody that antagonizes the bioactivities of B lymphocyte stimulator. Arthritis Rheum.

[11] (2018). Benlysta prescribing information. GlaxoSmithKline.

[12] Belimumab (Benlysta™) pregnancy registry. GlaxoSmithKline.

[13] Wazny K (2018). Applications of crowdsourcing in health: an overview. J Glob Health.

[14] Tucker JD, Day S, Tang W, Bayus B (2019). Crowdsourcing in medical research: concepts and applications. PeerJ.

[15] Truitt AR, Monsell SE, Avins AL, Nerenz DR, Lawrence SO, Bauer Z, Comstock BA, Edwards TC, Patrick DL, Jarvik JG, Lavallee DC (2018). Prioritizing research topics: a comparison of crowdsourcing and patient registry. Qual Life Res.

[16] Features. Amazon Mechanical Turk.

[17] Chandler J, Shapiro D (2016). Conducting clinical research using crowdsourced convenience samples. Annu Rev Clin Psychol.

[18] Sermo.

[19] (2020). Participation agreement. Amazon Mechanical Turk.

[20] (2021). Research Ethics Committee – Standard Operating Procedures. NHS Health Research Authority.

[21] (2016). Our Code of Practice for promotion and scientific engagement (prescription medicines). GSK.

[22] Chambers C, Johnson DL, Kiernan E (2018). Approach to evaluating pregnancy safety of anti-rheumatic medications in the OTIS MotherToBaby pregnancy studies: what have we learned?. Rheumatology (Oxford).

[23] Ling N, Lawson E, von Scheven E (2018). Adverse pregnancy outcomes in adolescents and young women with systemic lupus erythematosus: a national estimate. Pediatr Rheumatol Online J.

[24] Götestam Skorpen C, Hoeltzenbein M, Tincani A, Fischer-Betz R, Elefant E, Chambers C, da Silva J, Nelson-Piercy C, Cetin I, Costedoat-Chalumeau N, Dolhain R, Förger F, Khamashta M, Ruiz-Irastorza G, Zink A, Vencovsky J, Cutolo M, Caeyers N, Zumbühl C, Østensen M (2016). The EULAR points to consider for use of antirheumatic drugs before pregnancy, and during pregnancy and lactation. Ann Rheum Dis.

[25] Gelperin K, Hammad H, Leishear K, Bird ST, Taylor L, Hampp C, Sahin L (2017). A systematic review of pregnancy exposure registries: examination of protocol-specified pregnancy outcomes, target sample size, and comparator selection. Pharmacoepidemiol Drug Saf.

[26] Meissner Y, Fischer-Betz R, Andreoli L, Costedoat-Chalumeau N, De Cock D, Dolhain RJ, Forger F, Goll D, Molto A, Nelson-Piercy C, Özdemir R, Raio L, Rodríguez-García SC, Sciascia S, Wallenius M, Zbinden A, Zink A, Strangfeld A (2021). EULAR recommendations for a core data set for pregnancy registries in rheumatology. Ann Rheum Dis.

[27] Benevent J, Montastruc F, Damase-Michel C (2017). The importance of pharmacoepidemiology in pregnancy-implications for safety. Expert Opin Drug Saf.

[28] Goodman JK, Cryder CE, Cheema A (2013). Data collection in a flat world: the strengths and weaknesses of mechanical turk samples. J Behav Dec Making.

