# Supplementary material for: Identifying Barriers to Enrollment in Patient Pregnancy Registries: Building Evidence Through Crowdsourcing
Source: JMIR Form Res. 2022 May 25;6(5):e30573. doi: 10.2196/30573 (PMC9178445; doi:10.2196/30573)
Supplement: Multimedia Appendix 1 [file formative_v6i5e30573_app1.docx]

**Multimedia Appendix 1**

**Patient questionnaire**

**Instructions**

***Do not attempt this survey unless you meet all of the following criteria:***

1. a female between 18–55 years AND
2. have been diagnosed with Systemic Lupus Erythematosus (SLE) AND
3. have been pregnant within the past 2 years or are currently pregnant or trying to become pregnant now.

All others will be rejected.

Please answer the following questions based on your health condition and systemic lupus erythematosus (SLE) treatment options. This is an **anonymous** survey and should take no more than 10 minutes to complete.

**Please note:** Blank submissions, or not fully answering questions, will result in rejection of your submission and the worker possibly being blocked from future surveys. User comments may be left blank. Thank you for your hard work and happy turking!

**For support, contact us:** us.gcsp-sll@gsk.com

***Screening Questions***

**1. How old are you?**

*Please select one:*

- 18–25
- 26–35
- 36–45
- 46–55

**2. Where do you live?**

*Please select one:*

- USA
- Austria
- Belgium
- Canada
- France
- Germany
- Israel
- Portugal
- Slovakia
- Spain
- Sweden
- Other, specify below

Other Country: _____________

**3. Which, if any of the following health conditions has a doctor diagnosed you with?**

*Select all that apply:*

- Systemic Lupus Erythematosus (SLE)
- Other Lupus (cutaneous lupus, drug-induced lupus)

**4. Please rate the severity of your disease**

*Please select one:*

- Mild
- Moderate
- Severe

**5. Are you currently pregnant, planning a pregnancy now or recently been pregnant (in the past 2 years)?**

*Please select one:*

- No, I have not been pregnant in the past two years and I am not trying to become pregnant
- Currently pregnant
- Recently pregnant (past 2 years)
- Trying to become pregnant now

**6. Please indicate your current treatment options. Select all that apply**

*Select all that apply:*

- Non-steroidal anti-inflammatory drugs (NSAIDs) - such as Ibuprofen, Motrin®, Advil®, Aleve®, naproxen, etc.
- Corticosteroids - such as prednisone, Medrol®, etc.
- Antimalarials - such as hydroxychloroquine (Plaquenil®, Dolquine® and Quensyl®)
- Immunosuppressive agents - such as azathioprine (Imuran®, Azasan®), cyclophosphamide (Cytoxan®), cyclosporine (Neoral™, Sandimmune®), mycophenolate mofetil (CellCept®) and methotrexate (eg, Otrexup®, Rasuvo®, Rheumatrex® and Trexall®)
- Belimumab (Benlysta™)
- Rituximab (Rituxan™)

**7. If you were pregnant in the past two years, please indicate which treatment options you received while you were pregnant. Select all that apply**

*Select all that apply:*

- Non-steroidal anti-inflammatory drugs (NSAIDs) - such as Ibuprofen, Motrin®, Advil®, Aleve®, naproxen, etc.
- Corticosteroids - such as prednisone, Medrol®, etc.
- Antimalarials - such as hydroxychloroquine (Plaquenil®, Dolquine® and Quensyl®)
- Immunosuppressive agents - such as azathioprine (Imuran®, Azasan®), cyclophosphamide (Cytoxan®), cyclosporine (Neoral™, Sandimmune®), mycophenolate mofetil (CellCept®) and methotrexate (eg, Otrexup®, Rasuvo®, Rheumatrex® and Trexall®)
- Belimumab (Benlysta™)
- Rituximab (Rituxan™)

**8. Have you ever taken belimumab (Benlysta™)?**

*Please select one:*

- No
- Yes

***If you have taken Belimumab (Benlysta™)***

If you answered **Yes** to question 8, please answer the following, otherwise you may end the survey now and submit your response below.

**9. Are you currently taking belimumab (Benlysta™)?**

*Please select one:*

- No
- Yes

**10. If you are currently taking or have previously taken belimumab (Benlysta™), please indicate how long you were on it:**

*Please select one:*

- Have never taken belimumab (Benlysta™)
- Less than 1 year
- Between 1–2 years
- More than 2 years

**11. Did you use belimumab (Benlysta™) while pregnant or while trying to become pregnant?**

*Please select one:*

- No
- Yes - Used belimumab (Benlysta™) while pregnant
- Yes - Used belimumab (Benlysta™) while trying to become pregnant

**12. Did you stop taking belimumab (Benlysta™) due to a pregnancy or because you are trying to become pregnant?**

*Please select one:*

- Yes
- No

**13. Have you heard of the Belimumab (Benlysta™) Pregnancy Registry before?**

*Please select one:*

- Yes
- No

**14. If yes above, where did you learn about the Belimumab (Benlysta™) Pregnancy Registry?**

*Please select one:*

- Not applicable, never knew about the registry
- Friend or Family Member
- Doctor or other Health Care Provider
- Informational Brochure or Pamphlet
- Internet Search

***User Comments***

**Please provide any additional comments in the space below:**

___________________________

***Closing***

Thank you for your interest in participating in our research survey. Your time and information are greatly valued, as your responses will help us better understand patient needs. For more information, please visit the [Belimumab (Benlysta™) Pregnancy Registry](http://pregnancyregistry.gsk.com/belimumab.html). *The link will open in a new window and not submit your final response.*

# Rheumatologist questionnaire

***Screening Questions***

**S1) Do you prescribe belimumab?**

*Choose one answer:*

- Yes
- No (Screens out)

**S2) Do you treat women with systemic lupus erythematosus (SLE) who are or have been pregnant, or who are trying to get pregnant?**

*Choose one answer:*

- Yes
- No (Screens out)

***Survey Questions***

**Q1) How long have you been treating pregnant women with systemic lupus erythematosus (SLE)?**

*Please enter a numeric answer:*

_______ years

**Q2) What is the primary environment or setting that you practice in?**

*Choose one answer:*

- Hospital
- Academic Medical Center
- General Primary Care
- Private Practice
- Other

**Q3) When treating a woman who may be pregnant or who may be trying to conceive, what treatment options do you consider for various severities of SLE?**

*Per row, check all answers that apply:*

|  | Mild | Moderate | Severe |
| --- | --- | --- | --- |
| Non-steroidal anti-inflammatory drugs (NSAIDs) - such as Ibuprofen, Motrin®, Advil®, Aleve®, naproxen, etc. | 🞎 | 🞎 | 🞎 |
| Corticosteroids - such as prednisone, Medrol®, etc. | 🞎 | 🞎 | 🞎 |
| Antimalarials - such as hydroxychloroquine (Plaquenil®, Dolquine® and Quensyl®) | 🞎 | 🞎 | 🞎 |
| Immunosuppressive agents - such as azathioprine (Imuran®, Azasan®), cyclophosphamide (Cytoxan®), cyclosporine (Neoral™, Sandimmune®), mycophenolate mofetil (CellCept®) and methotrexate (eg, Otrexup®, Rasuvo®, Rheumatrex® and Trexall®) | 🞎 | 🞎 | 🞎 |
| Belimumab (Benlysta™) | 🞎 | 🞎 | 🞎 |
| Rituximab (Rituxan™) | 🞎 | 🞎 | 🞎 |
| Other | 🞎 | 🞎 | 🞎 |

**Q4) Are there any reasons why you would not prescribe belimumab during pregnancy?**

*Check all answers that apply:*

- None, I am willing to prescribe belimumab during pregnancy
- Unknown benefit/risk profile of belimumab in pregnancy
- Other treatment options are more desirable during pregnancy
- Disease is mild or symptoms tolerable
- Other

**Q5) Would you suggest a wash out period from belimumab prior to pregnancy? If yes, how long of a period?**

*Choose one answer:*

- No
- Yes – 0 to 30 days
- Yes – 30 to 60 days
- Yes – More than 60 days

**Q6) Have any of your patients expressed concerns about taking belimumab during pregnancy?**

*Check all answers that apply:*

- No concerns expressed
- Yes, patients are concerned with unknown safety profile of belimumab in pregnancy
- Yes, patient preference to use other treatment options
- Yes, patient desire to reduce or minimize all medication use while pregnant or trying to become pregnant
- Other

**Q7) Have you heard of the Belimumab (Benlysta™) Pregnancy Registry?**

*Choose one answer:*

- Yes
- No

**Q8) Would you refer your patients to the Belimumab (Benlysta™) Pregnancy Registry? If not, why?**

*Choose one answer:*

- Yes
- No

Reasons why not: ___________

**Q9) Over the course of your career, approximately how many women diagnosed with systemic lupus erythematosus (SLE) have you prescribed belimumab (Benlysta) and who were pregnant or recently had been, or who were trying to conceive?**

*Choose one answer:*

- Less than 5
- 5–10
- Greater than 1
