# Supplementary material for: Identifying Barriers to Enrollment in Patient Pregnancy Registries: Building Evidence Through Crowdsourcing
Source: JMIR Form Res. 2022 May 25;6(5):e30573. doi: 10.2196/30573 (PMC9178445; doi:10.2196/30573)
Supplement: Multimedia Appendix 2 [file formative_v6i5e30573_app2.docx]

**Multimedia Appendix 2.** Recruitment over time via the Amazon Mechanical Turk platform.
