# Supplementary material for: Identifying Barriers to Enrollment in Patient Pregnancy Registries: Building Evidence Through Crowdsourcing
Source: JMIR Form Res. 2022 May 25;6(5):e30573. doi: 10.2196/30573 (PMC9178445; doi:10.2196/30573)
Supplement: Multimedia Appendix 3 [file formative_v6i5e30573_app3.docx]

# Multimedia Appendix 3. Rheumatologists’ reasons for not referring patients to the belimumab registry.

| Alternative registry exists (n=2) |  |
| --- | --- |
|  | Einschluss in eigenes Schwangerschaftsregister Rhekiss |
|  | Other available registries |
| Unfamiliarity with BPR enrollment (n=4) |  |
|  | I am not familiar with the setup |
|  | Fill in myself? |
|  | I don’t know how |
|  | Possibly depending on set up |
| Unaware of BPR (n=2) |  |
|  | I don’ Know belimumab registry |
|  | Not aware of the pregnancy registry |
| Other (n=3) |  |
|  | Not available |
|  | Bureaucracy |
|  | Seems like a lot of work |

BPR, Belimumab Pregnancy Registry.
